# Dual-Antigen Subunit Vaccine Nanoparticles for Scrub Typhus

**DOI:** 10.3390/pathogens12121390

**Published:** 2023-11-25

**Authors:** Jaeyoung Park, Zhiwen Zhang, Tatyana Belinskaya, Alexandra N. Tsoras, Chien-Chung Chao, Le Jiang, Julie A. Champion

**Affiliations:** 1 School of Chemical and Biomolecular Engineering, Georgia Institute of Technology, 950 Atlantic Dr. NW, Atlanta, GA 30332, USA; jpark842@gatech.edu (J.P.); alextsoras@gmail.com (A.N.T.); 2 Henry Jackson Foundation for the Advancement of Military Medicine, 6720A Rockledge Dr, Bethesda, MD 20817, USA; zhiwen.zhang.ctr@health.mil (Z.Z.); tatyana.belinskaya.ctr@health.mil (T.B.); 3 Naval Medical Research Center, 503 Robert Grant Ave., Silver Spring, MD 20910, USA; ch3chao@gmail.com

**Keywords:** vaccine, scrub typhus, immunity, *Orientia*, nanoparticle

## Abstract

*Orientia tsutsugamushi* is the causative pathogen of scrub typhus, an acute febrile disease prevalent in the Asia–Pacific region that is spread to people through chigger bites. Despite the emerging threat, there is no currently available vaccine against *O. tsutsugamushi*. Here, we developed dual-antigen subunit vaccine nanoparticles using recombinant 47 kD and 56 kD proteins, which are immunogenic outer membrane antigens of *O. tsutsugamushi.* The biocompatible protein vaccine nanoparticles were formed via desolvation of r56 or r47E antigens with acetone, coating with an additional layer of the 56 kD protein, and stabilization with reducible homobifunctional DTSSP and heterobifunctional SDAD crosslinkers. The dual-antigen subunit vaccine nanoparticles significantly improved antigen-specific antibody responses in vaccinated mice. Most importantly, the dual-antigen nanoparticles coated with an additional layer of the 56 kD protein were markedly more immunogenic than soluble antigens or single-antigen nanoparticles in the context of cellular immune responses. Given the significance of cellular immune responses for protection against *O. tsutsugamushi*, these results demonstrate the potent immunogenicity of dual-layered antigen nanoparticles and their potential as a promising strategy for developing vaccines against scrub typhus.

## 1. Introduction

Scrub typhus is a neglected mite-borne disease caused by the obligate intracellular bacterium *Orientia tsutsugamushi* (*O. tsutsugamushi*), formerly known as *Rickettsia tsutsugamushi*. The disease was once thought to be restricted to the Tsutsugamushi Triangle but has become an emerging threat in new geographical areas, including the Middle East and South America [1]. Although the disease presents as an acute febrile illness that can potentially lead to life-threatening conditions, no vaccine is currently available for disease prevention. The increasing burden of disease has created an urgent demand for a vaccine against scrub typhus, but the development of a prophylactic vaccine has been hampered by the intracellular nature and antigenic heterogeneity of numerous *Orientia* strains [2,3,4,5]. While a combination of humoral and cellular immune responses synergizes to confer robust protection, T cell responses are indispensable for broad protection. As reported by many studies, serological antibodies from immunized or infected animals may lack cross-reactivity, but T cells from these animals are highly cross-protective against different *Orientia* strains [5]. Despite the significance of cellular immune responses, effective elicitation of T cell responses has yet to be achieved to date using various vaccine platforms, including formalin inactivated *O. tsutsugamushi* [6,7,8], DNA vectors [9,10], and soluble recombinant antigens [11,12,13,14].

In contrast to live *O. tsutsugamushi*, inactivated *O. tsutsugamushi* provided limited protection against homologous strains [5]. Vaccines targeting specific antigens have also been employed. In multiple studies, five proteins of *O. tsutsugamushi* with molecular sizes of 22 kD, 47 kD, 56 kD, 58 kD, and 100 kD were identified to be immunodominant in humans [15,16,17,18]. Among these, the 56 kD protein is the most immunodominant and can induce potent antibody responses, although it is strain-specific due to its high variability and, thus, provides limited cross-reactivity [19]. DNA vectors encoding the 56 kD protein induced immune responses against *O. tsutsugamushi*, but only 60% of challenged mice survived after four doses of the vaccine were given [10]. In lieu of DNA vectors, recombinant proteins, such as a truncated fragment of the 56 kD protein from the Karp strain, were also utilized in concert with adjuvants [11]. This approach resulted in moderate antibody and cellular immune responses but could not fully protect against infection. The 47 kD protein is another popular *Orientia* antigen recognized by the majority of patient sera and, unlike the 56 kD antigen, is highly conserved in 25 different strains of *O. tsutsugamushi* [19,20]. There was an attempt to use a recombinant 47 kD antigen fused to the 56 kD antigen (Sta56-47) as a vaccine [13]. Interestingly, mice administered with the recombinant Sta56-47 antigen mounted stronger humoral and cellular immune responses against the Karp strain of *O. tsutsugamushi* than those immunized with either the 56 kD or 47 kD antigen alone, which was correlated with superior protection and survival rate after challenge. This implied that the use of dual antigens could be more advantageous than a single antigen against *O. tsutsugamushi*.

Despite several efforts made in the formulation of soluble antigens as vaccine candidates, most soluble antigens are eliminated quickly, lack stability, and show poor endocytosis by antigen presenting cells (APCs) compared to nanoparticles (NPs) [21,22]. This often renders them less effective than the delivery of the antigen in NP form within the context of inducing immune responses. Additionally, nanotechnology has enabled the delivery of multiple different antigens and the design of platforms for effective presentation of antigens to immune cells [23,24]. For example, NPs can be externally decorated with antigens using chemical or biological conjugation methods to enhance the presentation of antigens to B cells, resulting in improved humoral immune responses [25]. Furthermore, NPs can act as adjuvants and enhance maturation, antigen processing, and cytokine release by APCs in addition to improving antigen uptake [26,27,28]. Biocompatibility must also be considered for vaccine development to avoid adverse effects. Polymers have been used for vaccine formulation to promote the stability of antigens and cellular uptake, but polymeric NPs require the use of biodegradable polymers to mitigate their toxicity [29,30,31,32]. Furthermore, polymers or their degradation products can induce unwanted immune responses, such as allergic reactions in the case of polyethylene glycol (PEG) [33,34,35,36] or acidification in the case of polylactic-co-glycolic acid (PLGA) [37,38]. In contrast to materials such as polymers or metal NPs, subunit vaccine NPs can be designed to safely stimulate the immune system while minimizing off-target immune responses by using biodegradable crosslinkers and only proteins as scaffolds of NPs, such as the antigen of interest [39,40].

Here, to enhance vaccine effectiveness, we developed subunit vaccine NPs using both recombinant 56 kD (r56) and 47 kD (r47E) antigens, given the cross-reactivity of r47E and strong immunogenicity of r56. The subunit vaccine NPs were formed via desolvation, and to augment the neutralizing activity of antibodies, the desolvated NPs were layered with an additional coating of the r56 antigen. Reducible 3,3′-dithiobis(sulfosuccinimidyl propionate) (DTSSP) and succinimidyl 2-((4,4′-azipentanamido)ethyl)-1,3′-dithiopropionate (SDAD) were exploited to stabilize and coat the subunit NPs with the r56 antigen, respectively, due to their biocompatibility and successful application for the development of vaccine NPs against influenza [41,42,43]. We hypothesized that the subunit NPs would improve both antibody and cellular immune responses, as we previously demonstrated greater immunogenicity of coated desolvated influenza antigen NPs compared to soluble antigens [41,44,45].

## 2. Materials and Methods

### 2.1. Production of Recombinant Antigens

The truncated (amino acids 80 to 456) recombinant major outer member protein antigen (r56) for the *O. tsutsugamushi* Karp strain was produced as previously described [46]. To produce the r47E antigen, the gene segment coding for amino acids 236–466 of the 47 kD antigen without the homologous protease domain [20] was cloned into plasmid vector pET28a (Novagen). The resultant plasmid was transformed and expressed in *E. coli* BL21(DE3) (ThermoFisher Scientific). Briefly, cells were grown in Overnight Express Instant TB medium (Novagen) containing 50 mg/mL kanamycin at 37 °C overnight. The cells were harvested, and the cell pellets were resuspended in 20 mM Tris and 0.15 M NaCl. The cells were lysed through sonication, followed by urea extraction performed sequentially using 2 M, 4 M, 6 M, and 8 M urea. The r47E inclusion bodies were collected with centrifugation, and the solubilized protein was collected. The solubilized protein was purified through a Ni-NTA column and eluted with a gradient of 25–400 mM imidazole. The concentrated elution fractions were pooled and further purified on an ion exchange column. The purified r47E was dialyzed sequentially against buffer (20 mM Tris-HCl, pH 8.0, 1 mM EDTA, 0.15 M NaCl) containing 6 M, 4 M, 2 M, 1 M, and, finally, no urea at 4 °C for refolding.

### 2.2. Rabbit Polyclonal Antibodies

Anti-sera containing polyclonal antibodies against the antigens were produced through rabbit injection with the corresponding recombinant proteins. Rabbit anti-Karp-r56 sera were produced by Covance Research Products (Denver, PA, USA). Rabbit anti-47E sera were produced by Pacific Immunology (Ramona, CA, USA).

### 2.3. Synthesis of Orientia Subunit Vaccine Nanoparticles

The subunit vaccine NPs were synthesized by desolvating r56, r47E, and a 1:1 molar mixture of r56–r47E. Prior to desolvation, r47E and r56 antigens were concentrated to 0.7–0.8 mg/mL with Amicon Ultra-0.5 Centrifugal Filter Units (3 kD MWCO). To desolvate the soluble proteins, 400 μL acetone was added dropwise at a rate of 1 mL/min to 100 μL of 0.6–0.8 mg/mL r56, r47E, or the 1:1 molar mixture of r56 and r47E antigens in storage buffer (20 mM Tris-HCl, pH 8.0, 1 mM EDTA, 1 mM DTT, 0.02% sodium azide) under constant stirring at 600 rpm. The desolvated NPs were then centrifuged at 14,000× *g* for 10 min at 10 °C. The NP pellets were resuspended in 500 μL storage buffer and stabilized with DTSSP crosslinker at a final concentration of 40.8 ng/μL while being stirred at 600 rpm for 1 h. After crosslinking, the NPs were centrifuged at 14,000× *g* for 10 min at 10 °C, and the NP pellets were resuspended in 200 μL storage buffer and sonicated for 1 sec on and 3 sec off at 50% amplitude for 1 min on ice. If the desolvated NPs were the final products, they were resuspended in 500 μL of phosphate buffered saline (PBS, pH 7.4).

To make the coated NPs, 200 μL of the desolvated NPs were incubated with SDAD crosslinker at a final concentration of 0.5 mM under constant stirring at 600 rpm for 30 min. Then, the crosslinking reaction was quenched with 10 μL of 1 M Tris-HCl at pH 8. The NPs were centrifuged at 14,000× *g* for 15 min at 10 °C, and the NP pellets were resuspended in 500 μL of 0.08 mg/mL r56 antigens in storage buffer. While being stirred at 600 rpm for 1 h, the photoactive diazirine group of the SDAD crosslinker was activated with UV light at 365 nm wavelength so that the diazirine group on the surface of the desolvated NPs could react with the r56 coat antigens. After crosslinking, the coated NPs were centrifuged for 20 min at 14,000× *g* at 10 °C, resuspended in 500 μL of PBS, and sonicated for 1 s on and 3 s off at 50% amplitude for 1 min on ice.

### 2.4. Characterization of Nanoparticles

The concentration of the *Orientia* subunit vaccine NPs was measured with the Bradford protein assay (ThermoFisher, Waltham, MA, USA) to evaluate NP percent yield, which was the ratio of NP mass to total mass of the antigens used multiplied by 100. The size and polydispesity index (PDI) of the NPs were assessed with dynamic light scattering (DLS) with a Malvern Zetasizer Nano ZS90. For each sample, three measurements of 15 runs were conducted for each sample at a scattering angle of 173° with a beam wavelength at 633 nm. For the measurement, a refractive index of 1.45 was used for proteins, and a refractive index of 1.33 with a viscosity of 0.8882 cP was used for PBS. The components of the NPs were evaluated with SDS-PAGE. For SDS-PAGE, 3 μg of the NPs was mixed with Laemmeli buffer solution containing β-mercaptoethanol and incubated for 5 min at 95 °C. Precision Plus Protein™ Dual Color standards (Biorad) and the NPs incubated with Laemmeli buffer were then separated by running a 12% SDS-PAGE gel for 80 min at 150 V in tris-glycine SDS running buffer. The proteins were stained with Coomassie Blue and imaged with the Gel Doc X + Gel Documentation System (Biorad).

### 2.5. ELISA for Analyzing Antigen Composition of Nanoparticles

Maxisorp 96-well immune assay plates (Nunc) were coated with 100 μL/well of 10^−6^–10^−1^ ng/μL *Orientia* subunit vaccine NPs or soluble antigen with 10-fold serial dilutions overnight at 25 °C. The plates were then washed three times with PBS containing 0.1% Tween-20 and blocked with 200 μL of 1% BSA in PBS for 1 h at 25 °C. After washing three times, rabbit anti-r56 and anti-r47E IgG antibodies were diluted at 1:3000 and added to the wells (100 μL/well). After incubation for 1 h at 25 °C, the plates were washed three times and incubated with 100 μL/well of goat anti-rabbit HRP-conjugated IgG (Southern Biotech) diluted at 1:5000 for 1 h at 25 °C. Each well was then washed three times and revealed with 50 μL of 1-Step Ultra TMB ELISA substrate solution (Thermo Fisher Scientific, Waltham, MA, USA) for 25 min. Color development was stopped by adding 50 μL/well of 2 N H_2_SO_4_ ELISA stop solution (Thermo Fisher Scientific). The absorbance at 450 nm was measured on a BioTek Synergy H4 micro plate reader.

### 2.6. Immunization of Mice

Five female C3HeB/FeJ mice at ~8 weeks old per group (The Jackson Laboratory, Bar Harbor, ME, USA) were administered with PBS (vehicle control), soluble proteins (r56 or r47E), or various nanoparticles through intramuscular injection. Each mouse received 10 µg total of soluble r56, soluble r47E, r56 NPs, r47E NPs, r47E NP + 56 Coat (9.4 µg of r47E + 0.6 µg of r56), r56/r47E Mix NP (4.5 µg of r56 + 5.5 µg of r47E), or r56/r47E Mix NP (~3.7 µg of r56 + ~4.5 µg of r47E) + r56 Coat (~1.8 µg) (5 µg in 25 or 35 μL saline into the thigh muscles of each of the hind limbs); the dose of antigens in each mixed NP was calculated with SDS-PAGE band intensity analysis using ImageJ 1.53k. All antigen preparations were checked for endotoxin levels using the Kinetic Chromogenic LAL Assay kit (Lonza, Basel, Switzerland), and when used for immunization, each mouse received endotoxin of less than 0.1 Endotoxin Units per injection (ranging from 0.024 to 0.093 EU/injection). The mice were immunized twice with a 3-week interval. Two weeks after the final immunization, all mice were euthanized for collection of serum and splenocytes for immune response assessment. All animal procedures were conducted under IACUC protocols approved by WRAIR/NMRC in compliance with the Animal Welfare Act and in accordance with the principles set forth in the “Guide for the Care and Use of Laboratory Animals,” Institute of Laboratory Animals Resources, National Research Council, National Academy Press, 2011.

### 2.7. ELISA for the Determination of Endpoint Titer

To determine antibody abundance in the serum samples from the immunized mice, an enzyme-linked immunosorbent assay (ELISA) was used to detect IgG specific to the r56 or r47E antigens using serially diluted serum samples and the procedure described previously [10]. The starting dilution of the serum was 1:100, followed by seven 4-fold dilutions (final dilution at 1:1,638,400). Area under the curve (AUC) values were calculated for each serum sample using Prism software (GraphPad, Boston, MA, USA) as described [47].

### 2.8. ELISPOT Assay for Cellular Immune Response

Splenocytes were isolated from immunized and control mice as previously described [48] and frozen in liquid nitrogen (1 × 10^7^ cells per vial) until use. The splenocytes were thawed rapidly in a 37 °C water bath and washed and resuspended in RPMI 1640 medium containing 2 mM L-glutamine, 10% heat-inactivated fetal bovine serum, and 1% penicillin and streptomycin. Subsequently, the splenocytes were incubated for approximately one hour at 37 °C in a CO_2_ incubator, while the mouse IFN-γ ELISpot kit (Mabtech, Cincinnati, OH, USA) was prepared according to the manufacturer’s instructions. After the incubation period, the cells were counted with the viability determined to be no less than 75%. A total of 2.5 × 10^5^ cells were seeded in each well of the ELISpot plate and stimulated with the control (PBS) or recombinant soluble antigens (r56 or r47E) at 5 µg/mL for 48 h. Detection of spots was performed based on the manufacturer’s guidance and scanned with an ELISpot reader (Advanced Imaging Devices GmbH, Straßberg, Germany) to determine the number of spots.

### 2.9. Statistical Analysis

For statistical comparison, one-way ANOVA was performed, followed by Turkey’s post hoc multiple comparison analysis when applicable. Statistical significance was determined as follows: (*) for *p* ≤ 0.05, (**) for *p* ≤ 0.01, (***) for *p* ≤ 0.001, (****) for *p* ≤ 0.0001. All data plotted with error bars represent mean values with standard deviation. The statistical analysis was performed with GraphPad Prism 9.

## 3. Results and Discussion

### 3.1. Optimization of Orientia Subunit Vaccine Nanoparticle Synthesis

**Figure 1:**
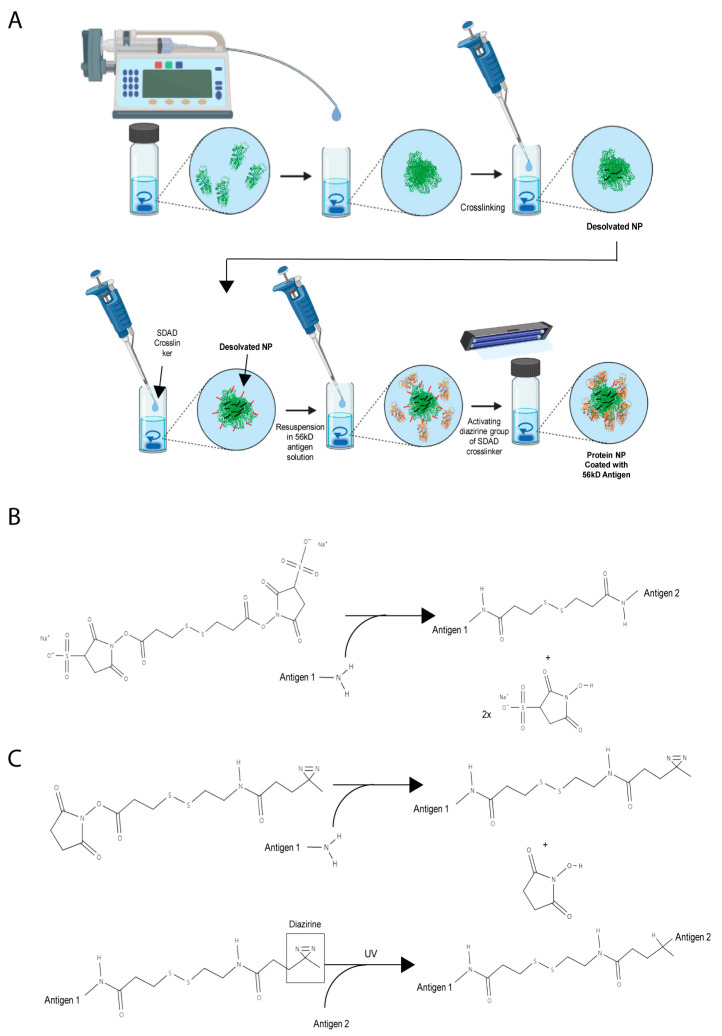
Schemes for (**A**) synthesis of *Orientia* subunit vaccine NPs and crosslinking of antigens with (**B**) DTSSP for NP stabilization and with (**C**) SDAD for NP coating.

**Figure 2:**
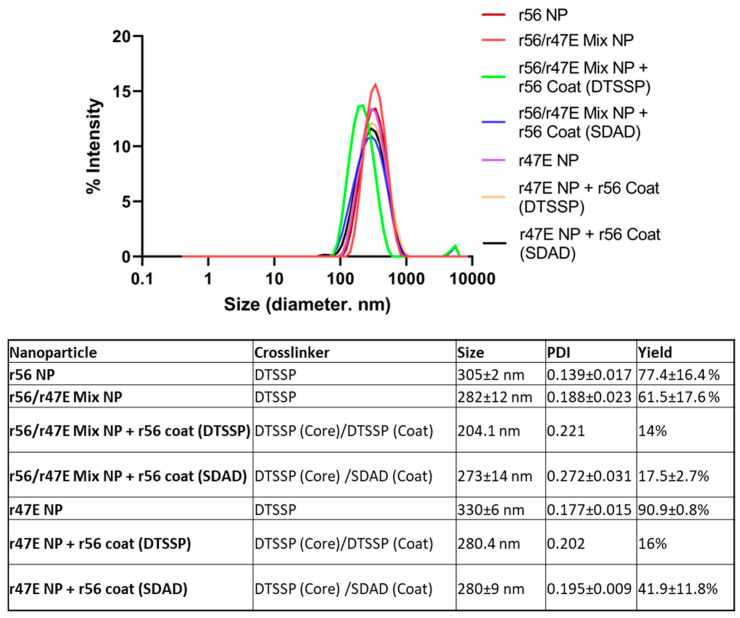
Physicochemical properties of *Orientia* subunit vaccine NPs.

Truncated recombinant 56 kD (amino acids 80–456, designated r56) and 47 kD (amino acids 236–466, designated r47E) antigens were overexpressed in *E.coli*, purified, and refolded. A 1:1 molar mixture of r56 and r47E antigens was desolvated with acetone to form the core for the *Orientia* subunit vaccine NPs (designated r56/r47E Mix NP) (Figure 1A). The size and yield of the desolvated NPs, defined as the percent ratio of NP mass to total mass of antigens used, were optimized, mainly by changing the volume of acetone addition, flow rate, and crosslinking procedure. Generally, when the flow rate and volume of acetone added increased, the size of the NPs decreased (Table S1). There was no clear trend observed for polydispersity of NP size distribution. A reducible crosslinker, DTSSP, was added to stabilize the NPs after desolvation by crosslinking amine groups of the antigens (Figure 1B). Due to the strong hydrophobic interactions between the antigens in acetone, crosslinking in the presence of acetone resulted in larger NPs than crosslinking after removal of acetone in most cases (Table S1A,B). Although the yield was generally low for most of the NPs, the solution of antigens turned cloudy when 400 μL of acetone or more was added to 100 μL of 0.4–0.5 mg/mL of soluble antigens. This indicates that a substantial amount of NPs was successfully formed at a 1:4 (%v:%v) or higher ratio of soluble antigen:acetone. After screening the combination of parameters for desolvation and crosslinking processes, the addition of 400 μL acetone at a rate of 1 mL/min to soluble antigens followed by crosslinking upon the removal of acetone was found to be the optimal condition for obtaining the smallest NPs with a size of 315 ± 15 nm. Nevertheless, the yield was only 10.6 ± 0.1%. To improve the yield of NPs, the 1:1 molar mixture of r47E and r56 antigens was concentrated from 0.4 to 0.5 mg/mL to 0.7 to 0.8 mg/mL, and the centrifugation temperature was decreased from 25 °C to 10 °C. These changes not only increased the yield but also improved the properties of the NPs. The yield increased to 61.5 ± 17.6%, and the size of the NPs decreased to 282 ± 12 nm with enhanced monodispersity (Figure 2). Using the same method, r47E-only and r56-only control NPs were prepared, resulting in sizes of 330 ± 6 nm and 305 ± 2 nm with 90.9 ± 0.8% and 77.4 ± 16.4% yields, respectively. As previously reported in other literature, smaller NPs tend to be generated by increasing the concentration of soluble protein, possibly due to the increased nucleation [49,50]. Interestingly, Tarhini et al. demonstrated that desolvated NPs became larger up to a certain mean size by increasing the concentration of soluble bovine serum albumin, but their size started to decrease at high protein concentrations [51]. This implies that NP nucleation and growth can largely be affected by protein concentration. Within the context of immunity and antigen delivery, the size of NPs is an important component of vaccine development. Small antigens or NPs (10–200 nm) can directly target the draining lymph nodes, which are the centers of the adaptive immune system, while large particles (500–2000 nm) cannot easily diffuse to lymphatic vessels [52,53,54,55,56,57]. Additionally, small NPs tend to show higher uptake of antigens by APCs. However, too small antigens or NPs (<5–10 nm) can be cleared quickly from the blood and simply ingested into the APCs with surrounding fluids by micropinocytosis, resulting in weaker activation of APCs than phagocytosis [56,58]. Therefore, it is important to maintain the size of NPs at less than 500 nm but larger than soluble antigens (~10 nm) to avoid micropinocytosis. Although it is ideal to achieve the size of NPs less than 100 nm for the direct transfer to lymphatic drainage, the size of NPs (200–350 nm) is within the acceptable size range for the effective activation of the immune system [53,59].

As reported by a previous study, the 47 kD antigen appeared to elicit stronger cellular immune responses than the 56 kD antigen, while the latter was more efficient at inducing humoral immune responses [60]. Therefore, we proposed to decorate the surface of NPs with the r56 antigen for maximizing recognition by B cell receptors, while the r47E antigen in the NP core could be internalized and processed by antigen presenting cells for the presentation of T cell epitopes. The r47E and r56/r47E Mix NPs were coated with the r56 antigen using the DTSSP or SDAD crosslinker. Homobifunctional DTSSP reacts with the amine groups of antigens (Figure 1B), whereas only the NHS-ester terminus of heterobifunctional SDAD can react with the amine groups of antigens (Figure 1C). The NPs were first reacted with the NHS-ester group of SDAD and then coated with r56 using adsorption. Upon exposure to UV light at 365 nm, the diazirine group of SDAD reacted with any amino acid of the adjacent r56 antigen coating (Figure 1A,C). Due to two differently controlled crosslinking reactions, use of the SDAD crosslinker can minimize the formation of NPs from soluble r56 coating protein or crosslinking between NPs. This approach also enables soluble r56 antigen to be reused for coating other NPs after separating the NP pellets from soluble r56 antigen. Furthermore, the yield increased by using the SDAD crosslinker compared to DTSSP (Figure 2). The final fabrication methods resulted in both r56/r47E Mix NPs + r56 Coat (SDAD) and r47E NPs + r56 Coat (SDAD) having a similar average diameter, approximately 280 nm.

**Figure 3:**
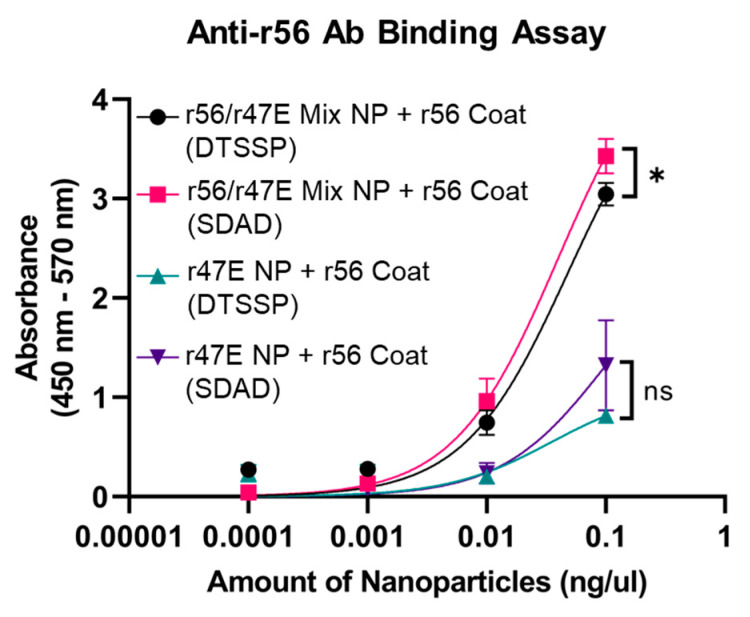
Comparison of *Orientia* subunit vaccine NPs coated with r56 protein with DTSSP and SDAD crosslinkers for binding avidity to anti-r56 antibody. (*) for *p* ≤ 0.05 and (ns) for statistically not significant.

To evaluate coating efficiency and retention of r56 recognition, an antibody binding assay was performed by employing ELISA with anti-r56 antibodies obtained from immunized rabbits. The r56/r47E Mix NPs coated with r56 using the SDAD crosslinker exhibited stronger binding to anti-r56 antibody than those using the DTSSP crosslinker (Figure 3). This suggests that SDAD crosslinking promoted efficient coating of NPs with the r56 antigen. Therefore, SDAD crosslinking was chosen for the final formulation of the coated dual-antigen vaccine NPs. Regardless of the crosslinker used, the r47E NPs exhibited reduced binding of anti-r56 antibodies and, likely, a less dense coating of r56. It is worth noting that r56 and r47E have 26 and 22 lysine residues, respectively (Figure S1), indicating that r47E has less conjugation sites than r56. This could lead to the more efficient coating of the r56/r47E Mix NPs than pure 47E NPs.

**Figure 4:**
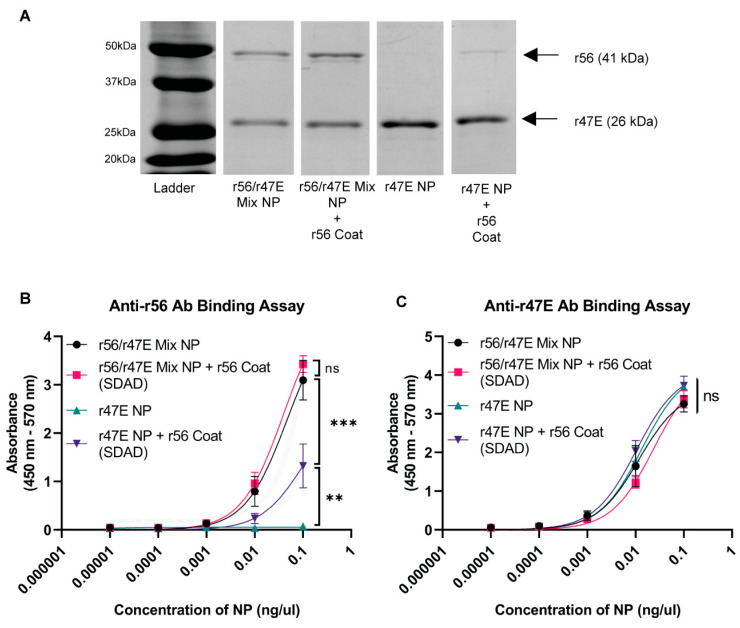
Antigen composition analysis for *Orientia* subunit vaccine NPs. (**A**) SDS-PAGE gel analysis of r56 and r47E antigens in *Orientia* subunit vaccine NPs. Binding avidity of *Orientia* subunit vaccine NPs coated with r56 protein with SDAD crosslinkers to (**B**) anti-r56 and (**C**) anti-r47E antibodies. (**) for *p* ≤ 0.01, (***) for *p* ≤ 0.001, and (ns) for statistically not significant.

The antigen composition and amount of r56 on the surface of the *Orientia* subunit vaccine NPs were evaluated with SDS-PAGE and an antibody binding assay. The NPs were broken up into r47E and r56 antigens with reducing crosslinkers and analyzed with SDS-PAGE. The r56 antigen was successfully detected from r56/r47E Mix NPs and r56/r47E Mix NPs + r56 Coat (Figure 4A), confirming that the mixed cores were indeed mixtures desolvated from both antigens. The band intensity of r56 from r56/r47E Mix NPs + r56 Coat (~45% r47E + ~37% r56 in mixed NP + ~18% r56 coated on NP by mass) was slightly greater than that of r56/r47E Mix NPs (~55% r47E + ~45% r56 by mass), and the r56 band was detected in r47E NPs + r56 Coat (~94% r47E + ~6% r56 by mass), confirming the presence of the r56 coat antigen on both types of NPs. The mass of r56 coated on r56/r47E Mix NPs was calculated by subtracting the band intensity of r56 in r56/r47E Mix NPs from that of r56 in r56/r47E Mix NPs + r56 Coat. The SDS-PAGE analysis also revealed a similar band intensity of r47E from both r56/r47E Mix NPs and r56/r47E Mix NPs + r56 Coat and a higher intensity of the r47E band for r47E NPs and r47E NPs + 56 Coat, as expected. Collectively, these data demonstrate that the NPs contain the proteins added during desolvation and coating.

Antibody binding was used to confirm the presence and accessibility of each antigen on the particle surface (Figure 4B). First, similar high binding of anti-r56 antibodies was observed on the surface of both r56/r47E Mix NPs and r56/r47E Mix NPs + r56 Coat. This indicates that there was a significant amount of r56 accessible on the mixed NP surface that was sufficiently folded to be recognized by the antibody. It also demonstrates that the r56 coating did not notably increase the amount of antibody accessible r56 on the particle surface. On the other hand, anti-r56 antibody bound significantly to r47E NPs + r56 Coat and not at all to r47E NPs. More anti-r56 antibodies bound to the mixed NPs (coated or uncoated) than to r47E NPs + r56 Coat, consistent with the lower r56 content observed with SDS-PAGE for r47E NPs + r56 Coat. Together, these data indicate that the r56 coating was functional and that the coating was not contiguous, making proteins from the underlying NP surface accessible. This aligns well with the anti-r47E antibody binding assay (Figure 4C), which demonstrated similarly strong binding to all NPs containing r47E. Since there was no reduction in anti-r47E binding observed with r56 coat or diluting the NP with r56, it suggests that steric hinderance of antibody binding to the surface of the NPs may limit the sensitivity of the assay.

### 3.2. In Vivo Assessment of Humoral Immunity of Orientia Subunit Vaccine Nanoparticles

**Figure 5:**
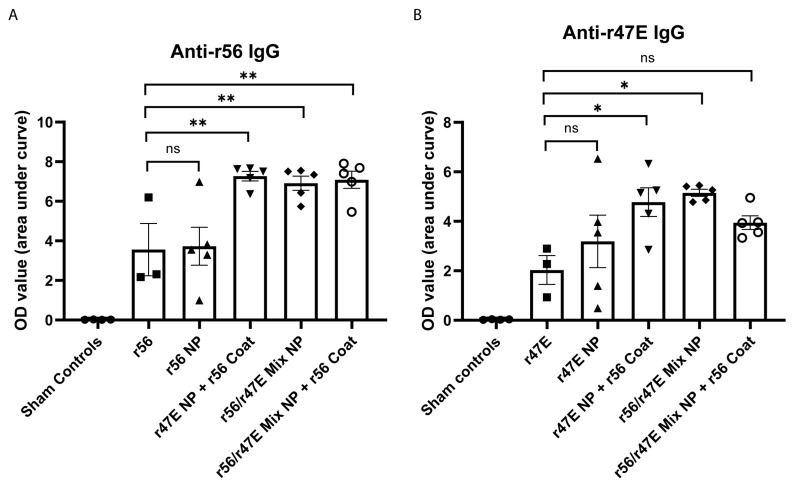
Abundance of antigen-specific IgG in serum collected from mice administered with the indicated soluble recombinant proteins or NPs to (**A**) r56 and (**B**) r47E antigens (each data point represents the average of duplicated ELISA assays performed on a serum sample of one individual mouse). (*) for *p* ≤ 0.05, (**) for *p* ≤ 0.01, and (ns) for statistically not significant.

To evaluate and compare the immunogenicity in vivo, we immunized C3HeB/FeJ mice intramuscularly with the same amount of either soluble antigens (r56 or r47E) or various NPs. ELISA data from serially diluted serum samples indicated that soluble antigens and NPs made from a single antigen induced similar levels of either r56- or r47E-specific antibodies, respectively (Figure 5A,B). Interestingly, the NPs containing dual antigens (r47E NP + r56 Coat, r56/r47E Mix NP with and without r56 Coat) induced significantly enhanced antibody responses against r56 (Figure 5A), although the amount of r56 antigens in the dual-antigen NPs is only approximately half of that in the single-antigen formulations (r56 and r56 NP); in Figure S2, the antibody binding assay revealed that soluble antigen r56 and r56 NP displayed stronger binding of anti-r56 antibody than r56/r47E Mix NP. Similar enhancement of humoral responses was also observed in the r47E ELISA data, where the dual-antigen NPs doubled the abundance of r47E-specific IgG (Figure 5B), though less antigens were present as compared to the single antigens (r47E and r47E NP). It also noteworthy that the effect of coating the NPs with antigens on humoral immune response was not evident when the coat protein (r56) was already incorporated in the NP (Figure 5). This is surprising given that r56/47E Mix and r56/r47E Mix NP + r56 Coat resulted in much stronger binding of anti-r56 antibody compared to r47E NP + r56 Coat (Figure 4A). A possible explanation is that the dose of r47E in the r56/r47E Mix NP + r56 Coat group was not high enough to induce statistically significant higher anti-r47E IgG titers than r47E as specified earlier. All together, these data suggest that incorporating dual antigens was more crucial for enhancing humoral immune responses than increasing the amount of antigens accessible on the surface.

### 3.3. In Vivo Assessment of Cellular Immunity of Orientia Subunit Vaccine Nanoparticles

**Figure 6:**
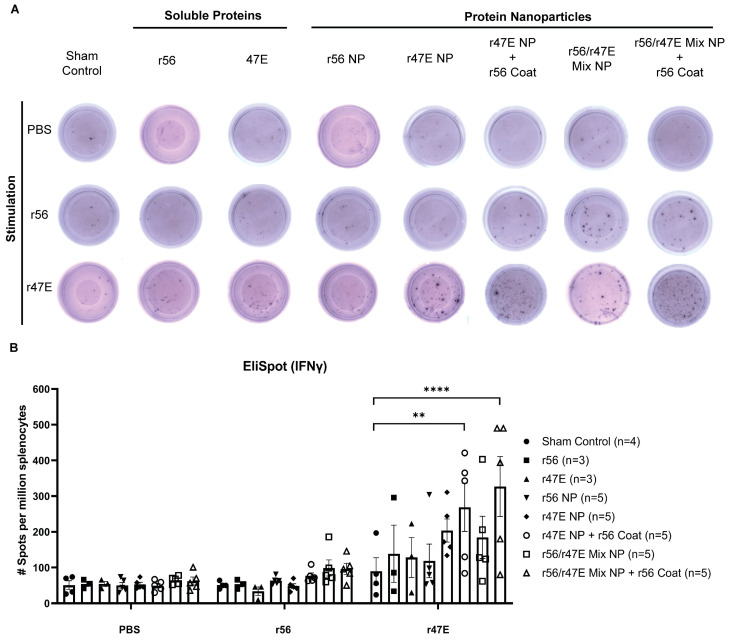
Significantly enhanced cellular immune responses in mice immunized with coated nanoparticles vs. soluble antigens. (**A**) The experiments were repeated three times, and representative ELISpot images are shown for IFNγ-secreting splenocytes after stimulating with PBS, r56, or r47E. (**B**) Quantification of ELISpot experiment in (**A**) and comparison of IFN-γ secreting splenocytes among control and immunized groups (the number of animals is indicated in the parentheses next to each group). (**) for *p* ≤ 0.01 and (****) for *p* ≤ 0.0001.

To assess the cellular immune responses in vaccinated mice, we isolated splenocytes and performed ELISpot assays to quantify IFNγ-secreting cells after incubation with r56 and r47E antigens as stimuli. In line with the previous notion that the 47 kD antigen is the dominant T cell antigen compared to other known *Orientia* antigens, including the 56 kD antigen [60], our ELISpot data clearly demonstrated that the cellular immunity in vaccinated mice is targeted toward the 47 kD antigen instead of the 56 kD antigen (Figure 6). Interestingly, soluble r47E and uncoated r47E NP antigens did not produce elevated levels of cellular immunity compared to the unimmunized control group. Increased cellular immune responses were only observed in the groups of mice immunized with NPs containing the 47 kD antigen, either pure or mixed, and coated with r56. While the effect of coating NPs with r56 on humoral immune responses was not pronounced, it significantly improved r47E-responsive cellular immune response. Although the mechanism underlying the improved cellular immune response by coating NPs with r56 remains elusive, the data clearly show that dual-antigen NPs are more effective than single-antigen NPs for eliciting immune responses.

## 4. Conclusions

Various strategies have been explored in an attempt to develop vaccines against *O. tsutsugamushi* [4,5]. Despite the continued effort, effective protection against *O. tsutsugamushi* has not been attained, especially due to an insufficient cellular immune response and poor heterologous protection against numerous strains. To address this issue and enhance vaccine effectiveness, we synthesized dual-antigen NPs consisting of immunodominant r47E and r56 antigens. NPs were fabricated consisting of r47E or a 1:1 mixture of r47E and r56 as a crosslinked NP core with an additional low-density coating of r56. Upon vaccination of mice, the dual-antigen NPs induced significantly higher levels of r47E- and r56-specific IgG antibodies than the single-antigen NPs or soluble antigen. Most importantly, the in vivo study revealed that the dual-antigen NPs induced potent cellular immune responses against the well-conserved 47 kD antigen when they were coated with the r56 antigen. Therefore, these data provide evidence for a feasible design of dual-antigen NP vaccine candidates against *O. tsutsugamushi* that can elicit both potent humoral and cellular immunity, serving as a very promising vaccine platform for scrub typhus. Future work will include an evaluation of challenge protection (both homologous and heterologous) from a panel of *Orientia* strains. Additionally, mixed NPs using r56 and/or r47E from several highly prevalent strains may be fabricated if further broadening of protection is needed.

## Data Availability

The data presented in this study are available on request from the corresponding author.

## Supplementary Materials

The following supporting information can be downloaded at: https://www.mdpi.com/article/10.3390/pathogens12121390/s1, Table S1: Optimization of desolvated NPs; Figure S1: Molecular weight of purified soluble r56 and r47E antigens analyzed by SDS-PAGE; Figure S2: Binding avidity of Orientia subunit vaccine NPs to r56 antibody.
