# Supplementary material for: Dual-Antigen Subunit Vaccine Nanoparticles for Scrub Typhus
Source: Pathogens. 2023 Nov 25;12(12):1390. doi: 10.3390/pathogens12121390 (PMC10745692; doi:10.3390/pathogens12121390)
Supplement: Supplementary file 1 [file pathogens-12-01390-s001.zip › pathogens-2711868-supplementary.pdf]

Dual-Antigen Subunit Vaccine Nanoparticles for Scrub Typhus

Jaeyoung Park[a], Zhiwen Zhang[b,c], Tatyana Belinskaya[b,c], Alexandra N. Tsoras[a,d], Chien-Chung Chao[c], Le Jiang[b,c,], Julie A. Champion[a,*]*

[a]School of Chemical and Biomolecular Engineering, Georgia Institute of Technology, 950 Atlantic Dr. NW, Atlanta, GA, 30332-2000, USA.
[b]Henry Jackson Foundation for the Advancement of Military Medicine, 6720A Rockledge Dr, Bethesda, MD 20817, USA.
[c]Naval Medical Research Center, 503 Robert Grant Ave. Silver Spring, MD, 20910, USA.
[d]2seventy bio, 60 Binney St, Cambridge, MA, 02142, USA.
[*]co-corresponding authors: julie.champion@chbe.gatech.edu (J.A.C) and le.jiang3.ctr@health.mil (L.J.)

A

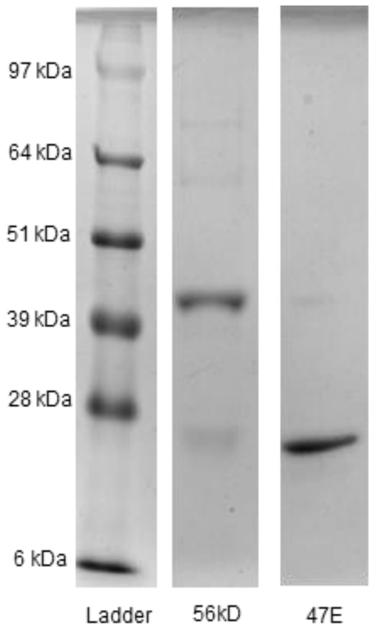

B

r56 (41 kD):
MTIAPGFRAEIGVMYLTNITAQVEEGKVKADSVGETKADS
VGGKDAPIRKRFKLTPPQPTIMPISIADRDFGIDIPNIPQQQ
AQAAQPQLNDEQRAAARIAWLKNCAGIDYRVKNPNDPN
GPMVINPILLNIPQGNPNPVGNPPQRANPPAGFAIHNHEQ
WRHLVVGLAALSNANKPSASPVKVLSDKITQIYSDIKPFA
DIAGIDVPDTSLPNSASVEQIQNKMQELNDLLEELRESFD
GYLGGNAFANQIQLNFVMPQQAQQQGQGQQQQAQATA
QEAVAAAAVRLLNGNDQIAQLYKDLVKLQRHAGIKKAME
KLAAQQEEDAKNQGEGDCKQQQGTSEKSKKGKDKEAE
FDLSMIVGQVKLYADVMITESVSI

r47E (26 kD):
NTVLEAVECLKKGEKIRRGMLNVMLNELTPELNENLGLK
QDQNGVLITEVIKEGSAAQCGIAPGDVITKFHDKAIKTGRD
LQVAVSSTMLNSEREVELLRNGKSMTLKCKIIANKGEDSE
QQSNDQSLVVNGVKFVDLTPDLVKKYNITSANNNGLFVL
EVSPNSSWGRYGLKMGLRPRDIILSVKRDDNKKDISVKTL
REIVTNIKHNEIFFTVQRGDRMLYIALPNINK

Figure S1. Molecular weights of purified soluble r56 and r47E antigens analyzed with (a) SDS-PAGE and calculated from (b) their sequences were 41 kD and 26 kD, respectively.

A

Method	Crosslinking right after desolvation but before centrifugation		
Agent	100% Acetone		
Volume	200 uL	400 uL	600 uL
0.25 mL/min		534.9 nm (n=1)<br>(yield: <1%)<br>PDI: 0.368	
0.5 mL/min	654.4 nm (n=1)<br>(yield: <1%)<br>PDI: 0.075	494.2 nm (n=1)<br>(yield: 54.6%)<br>PDI: 0.333	
1.0 mL/min	534.6 nm (n=1)<br>(yield: <1%)<br>PDI: 0.303	493.8 nm (n=1)<br>(yield: 55.3 %)<br>PDI: 0.261	469.8 nm (n=1)<br>(yield: <1%)<br>PDI: 0.295

B

Method	Crosslinking after desolvation and centrifugation		
Agent	100% Acetone		
Volume	200 uL	400 uL	600 uL
0.25 mL/min		1564 nm (n=1)<br>(yield: <1%)<br>PDI: 0.353	
0.5 mL/min	387.4 nm (n=1)<br>(yield <1%)<br>PDI: 0.211	345 ± 36 nm (n=3)<br>(yield: <1%)<br>PDI: 0.211 ± 0.033	
1.0 mL/min	1370 nm (n=1)<br>(yield: <1%)<br>PDI: 0.320	315 ± 15 nm (n=3)<br>(yield: 10.6 ± 0.1%)<br>PDI: 0.285 ± 0.034	327.9 nm (n=1)<br>(yield: <1%)<br>PDI: 0.341

Table S1. Optimization of desolvated NPs consisting of 1:1 molar mixture of r47E and r56 antigens. The nanoparticles were prepared by desolvating 100 μL of 0.4-0.5 mg/mL of the mixed antigens at 25℃. Flow rate and volume of acetone added to the antigen mixture were varied, while a DTSSP crosslinker was added (a) before or (b) after removal of acetone and resuspension in storage buffer.

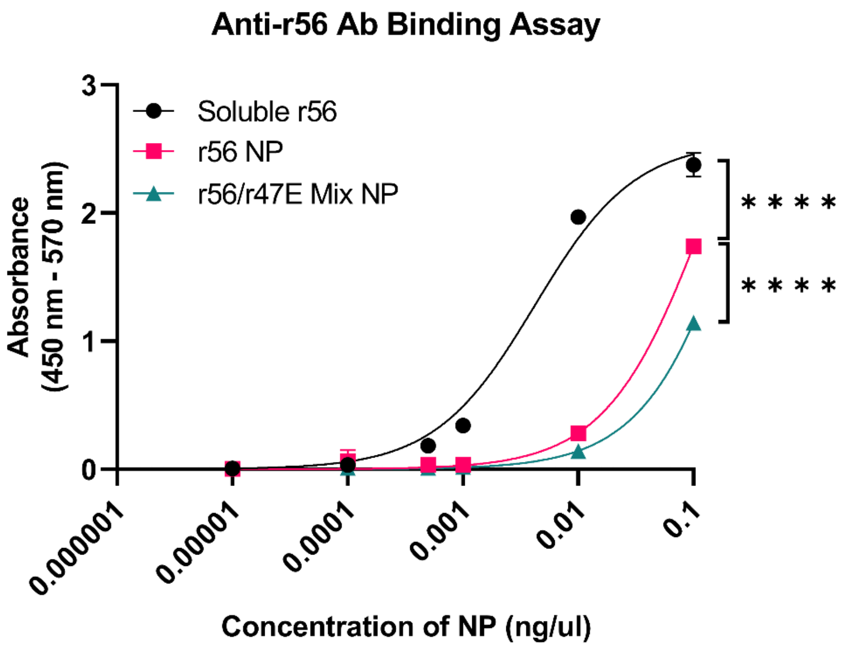

Figure S2. Binding avidity of *Orientia* subunit vaccine NPs and soluble r56 protein to rabbit anti-r56 antibody.
